# Editorial Comment on: Use and Persistence of Beta‐3 Adrenoceptor Agonists for the Treatment of Overactive Bladder

**DOI:** 10.1111/iju.70337

**Published:** 2025-12-27

**Authors:** Takanori Mochizuki

**Affiliations:** ^1^ Department of Urology, Interdisciplinary Graduate School of Medicine University of Yamanashi Chuo Yamanashi Japan

**Keywords:** LUTS, mirabegron, vibegron

The β3‐adrenoceptor agonists mirabegron and vibegron represent important therapeutic options for patients with overactive bladder (OAB), yet clinicians often face challenges in distinguishing how best to use each agent in daily practice. The present study provides timely real‐world data directly comparing these two agents at equivalent daily doses of 50 mg, offering one of the few opportunities to evaluate their effectiveness and persistence under routine clinical conditions [1].

A particularly notable finding is the relatively high discontinuation or switching rate observed among mirabegron users, with approximately 47.9% discontinuing the medication. The availability of vibegron as an alternative therapeutic agent likely contributed to this pattern, highlighting a prescribing dynamic that has not been previously captured in detail. The study thereby provides valuable insights into how the introduction of a second β3 agonist may influence clinical decision‐making, adherence, and treatment persistence. The inclusion of sex‐stratified analyses also enriches the study, offering more granular clinical information that may support individualized treatment selection.

Several randomized trials have compared mirabegron and vibegron in female OAB populations, generally demonstrating comparable overall efficacy, although vibegron may provide greater improvements in urinary frequency and urgency urinary incontinence in some cases [2, 3, 4]. However, although several comparative studies have focused on female patients, direct comparative evidence in male populations remains extremely limited, making the real‐world male‐predominant data presented in this study particularly informative.

As expected in retrospective real‐world research, several limitations should be acknowledged, including potential selection bias arising from clinicians' prescribing preferences and the absence of standardized criteria for determining treatment discontinuation. Confounding factors—such as comorbidities or prior treatment exposure—may also have influenced persistence outcomes. Nevertheless, the authors provide important observational evidence supporting real‐world treatment behaviors, including frequent switching from mirabegron to vibegron (40.7%) and the contribution of having multiple β3 agonist options to extended overall β3 agonist persistence.

The authors' earlier work reported favorable long‐term persistence with mirabegron before vibegron became available, and the current findings likely reflect the evolving therapeutic landscape. These results illustrate how the presence of multiple β3 agonist options may improve long‐term management by enabling treatment switching rather than treatment discontinuation. Such insights could help optimize OAB care, particularly given the known challenges with adherence and persistence across pharmacologic treatments for OAB.

Ultimately, while the present study does not establish superiority of one agent over the other, it sheds important light on real‐world utilization patterns and highlights the potential complementary roles of mirabegron and vibegron. Prospective randomized studies—including those specifically enrolling male patients—will be essential to confirm whether the observed persistence differences reflect intrinsic pharmacologic properties or prescribing behaviors.

Overall, this study contributes meaningful and clinically relevant evidence to the ongoing discussion on β3 agonist selection, reinforcing the importance of individualized treatment strategies and the value of having multiple therapeutic options available for patients with OAB.

## Author Contributions

**Takanori Mochizuki:** conceptualization, writing – review and editing.

## Conflicts of Interest

The author declares no conflicts of interest.

## Linked Articles

This article is linked to Wada et al. papers. To view this article, visit https://doi.org/10.1111/iju.70309.
